# Predictors of Higher Frequency of Atrial Fibrillation in Patients with Cardiac Resynchronization Therapy

**DOI:** 10.3390/medicina59122178

**Published:** 2023-12-15

**Authors:** Aleksandra Grbović, Siniša Pavlović, Vasko Žugić

**Affiliations:** Dedinje Cardiovascular Institute, Heroja Milana Tepića 1, 11000 Belgrade, Serbia; pavlosini@yahoo.com (S.P.); vaskozugicnk10@gmail.com (V.Ž.)

**Keywords:** heart failure, atrial fibrillation, cardiac resynchronization therapy

## Abstract

*Background and Objectives*: Cardiac resynchronization therapy (CRT) is one of the effective therapeutic options in the treatment of systolic heart failure (HF) with persistent symptoms. This prospective study was designed to determine whether CRT with biventricular pacing would reduce the risk of development of atrial fibrillation (AF) and to identify predictors for AF occurrence. *Materials and Methods*: The study population consisted of 126 patients, with a mean age of 63.8 ± 9.1 years, who were eligible for CRT with biventricular pacing. Inclusion criteria were left ventricular ejection fraction (LVEF) ≤ 35%, QRS duration ≥ 130 msec, and persistent HF symptoms of New York Heart Association (NYHA) II or III, despite optimal drug therapy. Patients were followed for a period of 24 months and were evaluated through clinical, electrocardiographic, and echocardiographic examination at baseline (prior to CRT implantation), as well as at 6 and 24 months post-implantation. At the end of follow-up, patients were divided into clinical responders and non-responders based on the following criteria: decrease in NYHA class ≥ I, increase in LVEF ≥ 10%, and reduction in QRS duration ≥ 20 msec. *Results*: At follow-up, CRT was associated with a significant increase in LVEF (20.6 ± 6.9% pre-implantation, 32.9 ± 9.3% 24 months after implantation; *p* < 0.001), reduction in left ventricular end-diastolic and end-systolic diameters, and decrease in QRS duration (167.6 ± 14.3 msec pre-implantation, 131.7 ± 11.7 msec 24 months after implantation; *p* < 0.001), while left atrial (LA) diameter was slightly increased (*p* = 0.070). The frequency of AF occurrence increased after two years of follow-up (52.4% to 56.9%, *p* < 0.001). Significant predictors of AF occurrence in our study population were response to CRT—AF more frequent in non-responders (B = 8.134; *p* < 0.001), LA diameter—AF more frequent with larger LA diameter (B = 0.813; *p* < 0.001), and coronary sinus (CS) lead position—AF more frequent with posterolateral in comparison with lateral CS lead position (B = 5.159; *p* = 0.005). *Conclusions*: The results of our study provide new data on AF predictors in patients with HF subjected to CRT. There remains a permanent need for new predictors, which might help in patient selection and improvement in response rate.

## 1. Introduction

Heart failure (HF) is a complex life-threatening clinical syndrome simply defined as the heart pumping an insufficient amount of blood to meet the body’s demands. The prevalence of HF is known to be 1–3% in the general adult population in developed countries [1]. According to the Global Burden of Disease Study, almost 65 million people were estimated to have been suffering from HF in 2017 [2]. With annual direct and indirect health care costs of up to EUR 25,000 per patient in developed countries, HF is recognized as a condition with high economic and social burden [1]. Due to the high public health importance of this condition, efforts have been directed towards developing efficient therapeutic options.

One of the breakthroughs in the treatment of systolic HF with persistent symptoms was the introduction of cardiac resynchronization therapy (CRT) in the 1990s. By simultaneous pacing of both the right and left ventricle, CRT offered effective restoration of left ventricular (LV) synchrony and improvement of LV mechanical function. Since the 1990s, CRT has emerged as a therapeutic option offering improvement in HF symptoms and quality of life, as well as a decrease in both HF-related hospitalizations and mortality rates [3,4]. Nowadays, CRT is regarded as a standard treatment in patients with New York Heart Association (NYHA) class II to class IV HF, which is based on strong evidence from several clinical trials [3,5,6,7,8,9,10]. In general, CRT is indicated in approximately one third of patients suffering from HF [11].

Atrial fibrillation (AF), the most common dysrhythmia [12], can attenuate the response to CRT in patients with HF mainly due to loss of atrioventricular synchrony and a higher risk for insufficient biventricular pacing [13,14,15,16]. A complex interplay exists between HF and AF, with AF being both the consequence and cause of HF. On one hand, HF can precipitate AF through structural and electrical remodeling of the atria. At the same time AF can reduce cardiac output due to irregular LV filling. In addition, it is also characterized by a negative influence on outcome in patients with HF [17,18]. It is estimated that approximately 10 million patients in Europe are affected by either paroxysmal or persistent AF, while it is expected that the number will rise to 14 million patients by 2030 [19,20]. The overall prevalence of AF in patients with HF is 25% [18] but, since the prevalence of AF depends on HF severity, AF is more common in higher NYHA stages (50% in NYHA class IV compared to 10% in NYHA class II) [21].

Since patients with HF and concomitant AF were mainly not included in the major randomized controlled trials of CRT, it is difficult to estimate the exact prevalence of AF in patients undergoing CRT implantation. However, there are some data showing that AF is present in 23% to 40% of patients receiving CRT at the time of implantation [21,22,23,24]. In addition, there is a possibility for new-onset AF that occurs after implantation in approximately one in five to one in four patients [25,26].

To further examine this issue, the following prospective study was undertaken with two aims set: to determine whether CRT with biventricular pacing would reduce the risk of development of AF and to identify predictors of AF in patients with HF and CRT.

## 2. Materials and Methods

This prospective study conducted at the Dedinje Cardiovascular Institute, Belgrade, Serbia, from 2019 to 2022, was approved by the Ethics Committee of the Medical Faculty, University of Belgrade No. 29/VI-15. Written informed consent was obtained from all study participants.

### 2.1. Study Subjects

The study population consisted of 126 patients with ischemic (N = 49) and nonischemic (N = 64) cardiomyopathy (CMP) or heart valve disease (N = 13). Inclusion criteria were as follows: left ventricular ejection fraction (LVEF) ≤ 35%, QRS duration ≥ 130 msec, and persistent HF symptoms of NYHA II or III, despite optimal drug therapy. Exclusion criteria were upgrade from cardiac resynchronization therapy pacemaker (CRT-P) to cardiac resynchronization therapy defibrillator (CRT-D), prior electrical cardioversion of AF and prior radiofrequency catheter ablation of pulmonary veins (pulmonary vein isolation).

### 2.2. Study Protocol

Patients were evaluated through clinical (NYHA class, type, and doses of diuretics), electrocardiographic (QRS duration), and echocardiographic (LV diameter, LV end-systolic dimension (ESD), LV end-diastolic dimension (EDD), LVEF, degree of mitral regurgitation (MR), and left atrial (LA) diameter) examination at baseline (before implantation of CRT-P), as well as at 6 and 24 months after CRT implantation. Immediately after implantation, patients were evaluated by chest roentgenogram (RTG) for coronary sinus (CS) lead position. The percentage of ventricular pacing was evaluated 24 months post-implantation. After 24 months, patients were divided into clinical responders and non-responders according to clinical (decrease in NYHA class ≥ I), echocardiographic (increase in LVEF ≥ 10%), and ECG (reduction in QRS duration ≥ 20 msec) improvement. Prior to CRT implantation, patients were assessed noninvasively for possible cardiac arrhythmias in order to exclude the presence of malignant cardiac arrhythmias. Monitoring was continued during the study period. If study participants experienced malignant cardiac arrhythmia, they were upgraded from cardiac resynchronization therapy pacemaker (CRT-P) to cardiac resynchronization therapy defibrillator (CRT-D) and excluded from the study.

### 2.3. Statistical Analysis

Depending on the type of variables and the normality of the distribution, results were presented as frequency (percent), median (range), and mean ± standard deviation. Statistical hypotheses were tested using *t*-test, Mann–Whitney test, ANOVA with repeated measures, Friedman test, or Wilcoxon test. Generalized linear mixed effects modeling approach within lme4 package for R was used to identify possible predictors of the dependent variables. Independent variables that were significant in univariate models were used as the independent variables in the multivariate model. All *p*-values less than 0.05 were considered significant. Statistical data analysis was performed using IBM SPSS Statistics 22 (IBM Corporation, Armonk, NY, USA) and R-4.0.0 software (4.0.0 April, 2020) (The R Foundation for Statistical Computing, Vienna, Austria).

## 3. Results

### 3.1. Baseline Characteristics

A total of 126 patients were enrolled in the study. Three patients prematurely ceased participation in the study due to lethal outcome after 3, 5, and 8 months from CRT implantation. Since a lethal outcome was considered a therapeutic failure, these patients were deemed to be non-responders.

The baseline characteristics of study participants are presented in Table 1. The average age of the study population was 63.8 ± 9.1 years (range 32–83 years). More than three quarters (77.8%) of the study participants were male.

In the majority of patients, nonischemic cardiomyopathy (50.8%) was the underlying cause of HF.

The pharmacotherapy of HF in study participants consisted of beta-adrenergic blockers (86.5%), angiotensin-converting enzyme inhibitors/angiotensin receptor blockers/angiotensin receptor/neprilysin inhibitors (90.5%), and mineralocorticoid receptor antagonists (77.0%). In addition to beta-adrenergic blockers, amiodarone (29.4%) and oral anticoagulants/non–vitamin K antagonist oral anticoagulants (58.7%) were used in the treatment of AF (Table 1).

After enrolment, a CRT device was implanted, with lateral or posterolateral CS lead position in more than 90% of patients and 6.3% with anterior CS lead position.

### 3.2. Clinical, Electrocardiographic, and Echocardiographic Effects of CRT

The response rate to CRT and the percentage of ventricular pacing are presented in Table 2. Almost 80% of the study participants were responders. The percentage of ventricular pacing after implantation was 96.4 ± 3.5% (range 85–99%). The difference in the percentage of ventricular pacing between responders (96.65 ± 3.33%) and non-responders (95.30 ± 4.09%) was not significant (*p =* 0.098).

The results from the clinical, electrocardiographic, and echocardiographic evaluation of the study participants during the follow-up period are presented in Table 3.

During follow-up, a significant improvement in the severity of HF was detected, both overall as well as when comparing in-between periods. This was reflected by an increase in the number of patients in stages I and II and a decrease in the number of patients in stage III. As expected, the observed improvement was accompanied by a significant increase in the use of “weaker” (thiazide) diuretics and a significant decrease in the use of “strong” (loop) diuretics.

Implantation of CRT device also resulted in a significant decrease in QRS duration (pre-implantation 167.6 ± 14.3 msec; 6 months 135.5 ± 10.4 msec; 24 months 131.7 ± 11.7 msec; *p* < 0.001 overall and between periods).

Both end-diastolic (EDD) and end-systolic (ESD) diameters of the LV significantly decreased (*p* < 0.001 overall and between periods) during the observed period, while LA diameter was slightly increased (*p =* 0.070).

The positive effect of CRT was also detected via significant improvement in LVEF (pre-implantation 20.6 ± 6.9%; 6 months 24.9 ± 7.8%; 24 months 32.9 ± 9.3%; *p* < 0.001 overall and between periods). Additionally, improvement in the severity of mitral regurgitation was observed. There was a significant increase in the number of patients with MR stage 1, accompanied by a decrease in the number of patients with MR stages 2 and 3 (*p* < 0.001 overall).

The results from the clinical, electrocardiographic, and echocardiographic evaluation were also analyzed separately for the groups of responders and non-responders and are presented in Table 4.

Significant differences between responders and non-responders were detected in the change in HF severity, with improvement being significantly stronger in responders compared to non-responders (after 24 months from CRT implantation *p =* 0.004; *p* value overall *p* < 0.001). The more pronounced improvement in responders was accompanied by more patients being treated with thiazide diuretics and lower doses of loop diuretics (furosemide) in the group of responders compared to non-responders (after 24 months from CRT implantation *p =* 0.003; *p* value overall *p* < 0.001).

On the other hand, QRS duration was significantly shortened (<0.001) in both responders (pre-implantation 167.2 ± 14.1 msec; after 24 months 131.3 ± 11.7 msec) and non-responders (pre-implantation 169.1 ± 15.3 msec; after 24 months 133.5 ± 11.9 msec).

Significant decrease in both EDD (*p* < 0.001) and ESD (*p* < 0.001) was detected in responders, while, in non-responders, the decrease was insignificant (EDD, *p =* 0.101; ESD, *p =* 0.058). At the same time, LA diameter was significantly increased (*p =* 0.022) in non-responders, while the change in LA diameter was negligible in responders.

The values of LVEF were similar at the beginning of this study in responders (20.7 ± 6.6%) and non-responders (20.4 ± 8.5%). Significantly higher values of LVEF were detected in responders compared to non-responders after 6 (25.8 ± 7.4% vs. 21.3 ± 8.7%, *p =* 0.013) and 24 months (35.0 ± 8.2% vs. 23.7 ± 8.4%, *p* < 0.001) from implantation.

### 3.3. Atrial Fibrillation

**Figure 1:**
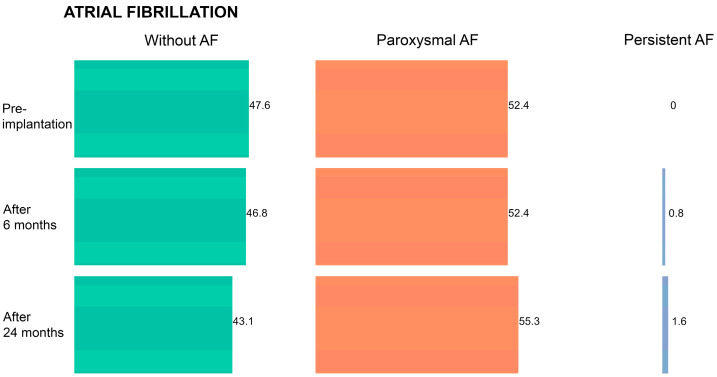
Frequency of atrial fibrillation (AF) in the overall patient cohort during the follow-up period.

During follow-up, the frequency of AF significantly increased among study participants (B = 2.058; *p* < 0.001) (Figure 1). The frequency of AF occurrence before CRT implantation was 52.4%, and it constantly rose during the follow-up period (53.2% after 6 months; 56.9% after 24 months).

When analyzed separately in groups of responders and non-responders, a constant rise in the frequency of AF occurrence was detected in both groups (Table 5). Although the absolute number of patients with AF in the group of non-responders appears to be decreasing in Table 5, the frequency of AF was actually rising due to dropouts in this group (three patients during the study period). The frequency of AF was significantly lower in the group of responders throughout the entire follow-up period (*p* < 0.001).

To identify significant predictors of AF in patients with an implanted CRT device, multi-variate mixed-effect regression analysis was performed with AF being the dependent variable (Table 6). All independent variables that were significantly associated (*p* < 0.001) with AF were included in the multivariate mixed-effect model, except for NYHA staging due to multicollinearity with EDD and CS lead position variables. Significant predictors of AF identified in this model were response to CRT with AF being more frequent in non-responders (B = 8.134; *p* < 0.001), CS lead position with AF being more frequent in patients with posterolateral in comparison to patients with lateral CS lead position (B = 5.159; *p =* 0.005), and LA diameter with AF being more frequent in patients with larger LA diameter (B = 0.813; *p* < 0.001).

## 4. Discussion

Heart failure is a global public health problem [1,2] that is more common in older age. While the prevalence of HF is 1% among people aged 45–55 years, it rises above 20% in people older than 85 years [27,28]. On the other hand, HF with preserved ejection fraction is more common in women, while HF with reduced ejection fraction is more common in men [29]. Similar characteristics were detected in our study participants. The average age of the patients was 63.8 ± 9.1 years and almost 80% were male. Their mean LVEF was 20.6 ± 6.9%. In approximately half of the study participants HF was of nonischemic etiology, while ~40% had ischemic heart disease. These data are in accordance with data from other studies, in which ischemic heart disease has been identified as one of the most common solitary causes of HF [1,30].

After initial evaluation, CRT devices were implanted to study participants. In most patients in our study, CS lead position was lateral (48.4%) or posterolateral (45.2%), which are known to be associated with more efficient CRT compared to anterior position, as presented in several observational studies [31,32,33].

Following implantation, the effects of CRT were evaluated 6 and 24 months later. The response rate to CRT was almost 80% at 24 months post-implantation. This figure is higher than the rate observed in other studies, which is reported to be up to 70% [6,34,35,36].

During the follow-up period, significant improvement was detected in all the evaluated parameters except for the LA diameter, which did not change significantly during the period of 24 months.

While 110 patients were classified in NYHA III at the time of enrolment, after 24 months, only one patient was classified in NYHA stage III. At the same time, the number of patients increased from 16 to 83 in NYHA stage II and from 0 to 39 in NYHA stage I. Such improvement was expected, given that many studies, including the very first clinical trials, have already demonstrated the effectiveness of CRT [5,6].

One of the easily quantifiable parameters in CRT is QRS duration, which, when prolonged, suggests the presence of electrical dyssynchrony resulting in decreased LV function [37,38]. Already, at 6 months after CRT implantation, QRS duration was significantly shortened in our study population (pre-implantation 167.6 ± 14.3 msec; after 6 months 135.5 ± 10.4 msec; *p* < 0.001), leading to better LV function as evidenced by an increase in LVEF (~12% during the study period). As expected, functional improvement was accompanied with structural changes, since shortening of QRS duration is known to correlate positively with LV reverse remodeling [39].

It is known that cardiac remodeling, clinically manifested as LV dilatation, can be reversed with CRT. Most frequently, reverse remodeling is evaluated by reduction in LV size and LVEF improvement [6,8,9]. In our study, both EDD (pre-implantation 66.1 ± 7.1 mm; after 24 months 62.2 ± 7.4 mm) and ESD (pre-implantation 51.2 ± 8.0 mm; after 24 months 47.7 ± 7.6 mm) significantly (*p* < 0.001) decreased during the study period. Improvement in cardiac structure due to CRT can also result in mitral annulus tightening, leading to less severe MR [37,40]. This effect was detected in our study as well. During the follow-up period of 24 months, the number of patients with severe MR decreased. There was a reduction in the number of patients with MR grade 3 from 21 to 1 (already observed after 6 months from CRT implantation), while patients with MR grade 2 decreased from 55 to 19.

With a decrease in LV size, improvement in LVEF, and reduction in MR, it was reasonable to expect reduction in LA size, previously reported in other similar studies [41,42]. In the study of Badran et al., a significant decrease in LA size of approximately 5% was detected in a group of patients who responded to CRT just after 3 months from implantation [43]. However, in our overall study population (pre-implantation 44.8 ± 4.7 mm; after 24 months 45.3 ± 5.0 mm), as well as in the group of responders (pre-implantation 44.5 ± 4.8 mm; after 24 months 44.8 ± 5.0 mm) and non-responders (pre-implantation 45.9 ± 3.8 mm; after 24 months 47.3 ± 4.7 mm) separately, no improvement was detected. Instead of reduction, an increase in LA diameter (significant in non-responders, *p =* 0.022) was observed.

In addition, the frequency of atrial fibrillation in our study population did not decrease over time. Such a result might be anticipated since studies suggest that there exists a relationship between AF frequency and increased LA size [44]. At baseline (before CRT implantation), 47.6% of the patients did not have AF and the remaining 52.4% were diagnosed with paroxysmal AF. At 24 months post-implantation, 43.1% of the study participants did not have AF, whereas 55.3% were suffering from paroxysmal AF and 1.6% from persistent AF. The frequency of AF occurrence was significantly higher in the group of non-responders compared to responders (*p* < 0.001) throughout the entire follow-up period.

As previously mentioned, AF can reduce the response to CRT in patients with HF. The attenuated response to CRT is mainly caused by loss of atrioventricular synchrony, which may result in inadequate biventricular capture [13,14,15,16]. In order to improve the management of patients who undergo CRT and may develop AF, efforts have been focused on identifying predictors of AF which might help in maximizing the therapeutic effects of CRT. According to the results of the study by Nedios et al., patients with persistent AF, dilated LA, and advanced age are at higher risk for developing permanent AF during CRT [45]. In case of new-onset AF, Sade et al. suggest that LA functional improvement is recognized as an independent predictor of AF-free survival in CRT patients and is, therefore, essential for maintaining favorable long-term outcomes in these patients [46]. In the same study, the authors also acknowledge that new-onset AF is associated with a decline in CRT effects, including effects on LV volumes, EF, and MR.

Significant predictors of AF in our study population, identified with multivariate mixed-effect regression analysis, were the following: response to CRT—higher frequency of AF in non-responders (B = 8.134; *p* < 0.001), LA diameter—higher frequency of AF in patients with larger LA diameter (B = 0.813; *p* < 0.001), and CS lead position—higher frequency of AF in patients with posterolateral in comparison with lateral CS lead position (B = 5.159; *p =* 0.005).

As mentioned above, there is a relationship between increased LA size and AF. However, based on the results of several studies, it is not completely clear if the increase in LA size is the cause or the consequence of AF [47,48]. In our study population, increased LA size was identified as a predictor of AF. Similar results have been reported in a review article by Inciardi and Rossi, according to which a 30% increase in LA volume was independently associated with a 43% increase in the risk of developing AF [44].

Non-response to CRT was another predictor of AF identified in regression analysis. Such a result can be explained by the absence of reverse LV remodeling and the lack of decrease in MR severity in the group of non-responders. Lack of both reverse LV remodeling and decrease in MR severity affects LA hemodynamics and size. The effect of LV remodeling on LA function was described in a meta-analysis by Bytyçi et al., who reported improvement in LA hemodynamics and function in patients responding to CRT [49]. On the other hand, it is known that MR also works in favor of AF. In a study by Zhao et al., MR was identified as an independent predictor of AF recurrence after ablation in patients with persistent AF, with the rate of recurrence depending on MR severity [50].

The third predictor of AF identified in our study population was the CS lead position. It was found that AF was more frequent in patients with posterolateral in comparison with lateral CS lead position. During the procedure of CRT implantation, the most challenging part is the placement of the CS lead. It is affected by the variable anatomy of the coronary venous system and it may also be demanding in cases where patients have previously undergone cardiac surgical procedures, aorto-coronary venous grafting, or CS lead extractions [51]. In everyday practice, the lateral CS lead position is preferred, since it is associated with better clinical outcomes [31,32,33]. However, not so many studies have compared lateral and posterolateral positions. In a study by Behon et al., which included a retrospective database of almost 2100 patients, lateral CS lead position was found to be superior compared to posterolateral position in terms of improvement in echocardiographic response and long-term mortality [52].

With prevalence of up to 50% [21], AF is an important obstacle to effective CRT in patients suffering from HF. To avoid CRT therapeutic failure, it is worth looking for predictors which would identify patients more prone to AF. Such patients might be subjected to some additional therapeutic measures, such as ablation, to increase success rate in CRT [14,53,54]. In our study, we have managed to identify three significant predictors of AF in patients with HF undergoing CRT. However, our study has several limitations, including single-center design and limited sample size. In addition, there is a growing demand for re-evaluation of the criteria of therapeutic response to CRT used in our study. A growing number of authors suggest that the current criteria used to evaluate therapeutic response to CRT should be improved to better represent response or non-response to CRT [55,56,57]. Another limitation that has to be mentioned is that two predictors of AF (response to CRT and CS lead position) identified in our study are post-CRT parameters, what limits the possibility of using these predictors in patient selection, and improvement in response rate. On the other hand, our study was a prospective study with a negligible dropout rate (three patients in the group of non-responders), thus enabling reliable follow-up during the entire study period.

## 5. Conclusions

The results of our study provide new data on AF predictors in patients with HF subjected to CRT. Due to lack of studies on CRT recruiting patients with AF, there is a demand for new studies with bigger sample sizes to identify new predictors. Studies on genes and genetic variations making patients more prone to AF in HF would be of particular interest. By identifying patients more prone to AF prior to CRT implantation, it would be possible to gain maximum benefit from CRT, a treatment that has thus far offered significant improvement in the management of patients with HF.

## Figures and Tables

**Table 1:** Baseline characteristics of study population (n = 126).

Age, years (x^−^ ± SD)	63.8 ± 9.1
Sex, N (%)	
male	98 (77.8%)
female	28 (22.2%)
CMP, N (%)	
nonischemic	64 (50.8%)
ischemic	49 (38.9%)
heart valve disease	13 (10.3%)
Pharmacotherapy N (%)	
HF	
beta-adrenergic blockers	109 (86.5%)
ACE-I/ARB/ARNI	114 (90.5%)
MRA	97 (77.0%)
AF	
Amiodarone	37 (29.4%)
OAC/NOAC	74 (58.7%)
CS lead position, N (%) lateral anterior posterolateral	61 (48.4%) 8 (6.3%) 57 (45.2%)

Abbreviations: ACE-I = angiotensin-converting enzyme inhibitors; AF = atrial fibrillation; ARB = angiotensin receptor blockers; ARNI = angiotensin receptor/neprilysin inhibitors; CMP = cardiomyopathy; CS = coronary sinus; HF = heart failure; MRA = mineralocorticoid receptor antagonists; NOAC = non–vitamin K antagonist oral anticoagulants; OAC = oral anticoagulants; SD = standard deviation.

**Table 2:** Response rate to CRT and percentage of ventricular pacing.

Response to CRT, N (%) R nonR	100 (79.4%) 26 (20.6%)	
Ventricular pacing, % (x^−^ ± SD)		
Total	96.4 ± 3.5	
R	96.65 ± 3.33	*p =* 0.098
nonR	95.30 ± 4.09	

Abbreviations: CRT = cardiac resynchronization therapy; NonR = non-responder; R = responder; SD = standard deviation.

**Table 3:** Clinical, electrocardiographic, and echocardiographic evaluation of the study cohort at baseline and at 6-month and 24-month follow-up.

	Pre-Implantation	6 Months	24 Months	*p* Overall	*p* Pre-Implant. vs 6 m	*p* Pre-Implant. vs 24 m	*p* 6 m–24 m
NYHA, N (%) I II							
	0 (0.0%)	3 (2.4%)	39 (31.7%)				
	16 (12.7%)	116 (93.5%)	83 (67.5%)	<0.001	<0.001	<0.001	0.014
III	110 (87.3%)	5 (4.0%)	1 (0.8%)				
Diuretic, N (%)							
thiazide	0 (0.0%)	3 (2.4%)	58 (47.2%)				
furosemide							
40 mg	79 (62.7%)	99 (79.8%)	57 (46.3%)	<0.001	0.005	<0.001	<0.001
80 mg	23 (18.3%)	20 (16.1%)	7 (5.7%)				
250 mg	21 (16.7%)	2 (1.6%)	1 (0.8%)				
500 mg	3 (2.4%)	0 (0.0%)	0 (0.0%)				
QRS msec (x^−^ ± SD)	167.6 ± 14.3	135.5 ± 10.4	131.7 ± 11.7	<0.001	<0.001	<0.001	<0.001
EDD mm (x^−^ ± SD)	66.1 ± 7.1	64.3 ± 6.7	62.2 ± 7.4	<0.001	<0.001	<0.001	<0.001
ESD mm (x^−^ ± SD)	51.2 ± 8.0	49.6 ± 7.8	47.7 ± 7.6	<0.001	<0.001	<0.001	<0.001
LVEF % (x^−^ ± SD)	20.6 ± 6.9	24.9 ± 7.8	32.9 ± 9.3	<0.001	<0.001	<0.001	<0.001
LA mm (x^−^ ± SD)	44.8 ± 4.7	45.0 ± 4.7	45.3 ± 5.0	0.070			
MR, N (%)							
1	50 (39.7%)	94 (75.8%)	103 (83.7%)				
2	55 (43.7%)	29 (23.4%)	19 (15.4%)	<0.001	<0.001	<0.001	<0.001
3	21 (16.7%)	1 (0.8%)	1 (0.8%)				

Abbreviations: EDD = end-diastolic diameter; ESD = end-systolic diameter; LA = left atrium; LVEF = left ventricular ejection fraction; MR = mitral regurgitation; NonR = non-responder; NYHA = New York Heart Association; R = responder.

**Table 4:** Clinical, electrocardiographic, and echocardiographic evaluation in responders and non-responders.

	Group	Pre-Implantation	6 Months	24 Months	*p*-Value Overall
NYHA, N (%) I II III	R	0 (0.0%) 10 (10.0%) 90 (90.0%)	3 (3.0%) 94 (94.0%) 3 (3.0%)	37 (37.0%) 63 (63.0%) 0 (0.0%)	<0.001
	nonR	0 (0.0%) 6 (23.1%) 20 (76.9%)	0 (0.0%) 22 (91.7%) 2 (8.3%)	2 (8.7%) 20 (87.0%) 1 (4.3%)	<0.001
	*p*-value *	0.076	0.150	0.004	
Diuretic. N (%) thiazide furosemide 40 mg furosemide 80 mg furosemide 250 mg furosemide 500 mg	R	0 (0.0%) 67 (67.0%) 18 (18.0%) 13 (13.0%) 2 (2.0%)	1 (1.0%) 84 (84.0%) 14 (14.0%) 1 (1.0%) 0 (0.0%)	53 (53.0%) 43 (43.0%) 3 (3.0%) 1 (1.0%) 0 (0.0%)	<0.001
	nonR	0 (0.0%) 12 (46.2%) 5 (19.2%) 8 (30.8%) 1 (3.8%)	2 (8.3%) 15 (62.5%) 6 (25.0%) 1 (4.2%) 0 (0.0%)	5 (21.7%) 14 (60.9%) 4 (17.4%) 0 (0.0%) 0 (0.0%)	<0.001
	*p*-value *	0.029	0.377	0.003	
QRS msec (x^−^ ± SD)	R	167.2 ± 14.1	135.4 ± 10.3	131.3 ± 11.7	<0.001
	nonR	169.1 ± 15.3	136.1 ± 11.2	133.5 ± 11.9	<0.001
	*p*-value *	0.562	0.777	0.424	
EDD mm (x^−^ ± SD)	R	66.3 ± 7.2	64.3 ± 6.8	61.7 ± 7.3	<0.001
	nonR	65.4 ± 6.8	64.5 ± 6.8	64.2 ± 7.4	0.101
	*p*-value *	0.557	0.887	0.072	
ESD mm (x^−^ ± SD)	R	51.3 ± 8.0	49.6 ± 7.7	47.4 ± 7.3	<0.001
	nonR	50.7 ± 8.3	49.7 ± 8.1	49.0 ± 8.7	0.058
	*p*-value *	0.762	0.995	0.372	
LVEF % (x^−^ ± SD)	R	20.7 ± 6.6	25.8 ± 7.4	35.0 ± 8.2	<0.001
	nonR	20.4 ± 8.5	21.3 ± 8.7	23.7 ± 8.4	<0.001
	*p*-value *	0.910	0.013	<0.001	
LA mm (x^−^ ± SD)	R	44.5 ± 4.8	44.6 ± 4.8	44.8 ± 5.0	0.346
	nonR	45.9 ± 3.8	46.3 ± 4.0	47.3 ± 4.7	0.022
	*p*-value *	0.197	0.123	0.017	
MR. N (%) 1 2 3	R	39 (39.0%) 43 (43.0%) 18 (18.0%)	79 (79.0%) 21 (21.0%) 0 (0.0%)	86 (86.0%) 14 (14.0%) 0 (0.0%)	<0.001
	nonR	11 (42.3%) 12 (46.2%) 3 (11.5%)	15 (62.5%) 8 (33.3%) 1 (4.2%)	17 (73.9%) 5 (21.7%) 1 (4.3%)	<0.001
	*p*-value *	0.572	0.076	0.139	

Abbreviations: EDD = end-diastolic diameter; ESD = end-systolic diameter; LA = left atrium; LVEF = left ventricular ejection fraction; MR = mitral regurgitation; NonR = non-responder; NYHA = New York Heart Association; R = responder. * *p*-value of intergroup differences.

**Table 5:** Frequency of atrial fibrillation in responders and non-responders during the follow-up period.

	Group	Pre-Implantation	6 Months	24 Months	*p*-Value Overall
Atrial fibrillation N (%)	R	45 (45.0%)	46 (46.0%)	50 (50.0%)	<0.001
	nonR	21 (80.8%)	20 (83.3%)	20 (87.0%)	<0.001
	*p*-value *	0.001	0.001	0.001	

Abbreviations: NonR = non-responder; R = responder. * *p*-value of intergroup differences.

**Table 6:** Generalized linear mixed-effect regression model with atrial fibrillation being the dependent variable.

	Univariable		Multivariable	
	B	*p*	B	*p*
Age	0.099	0.401		
Sex (male vs. female)	24.360	<0.001	2.263	0.061
Response to CRT (non-responder/responder)	25.683	<0.001	8.134	<0.001
Ventricular pacing	−1.674	<0.001	−0.366	0.277
CMP non-ischemic ischemic heart valve disease				
	ref 23.979 24.472	<0.001 <0.001	2.194 2.633	0.194 0.425
CS lead position lateral anterior posterolateral	ref 24.261 25.143	<0.001 <0.001	1.650 5.159	0.566 0.005
NYHA	2.481	<0.001		
Diuretic	1.581	0.154		
QRS	0.050	0.248		
EDD	0.167	<0.001	0.069	0.497
ESD	0.082	0.386		
LA	3.001	0.005	0.813	<0.001
MR	1.175	<0.001	0.887	0.313
LVEF	−0.030	0.703		

Abbreviations: CMP = cardiomyopathy; CRT = cardiac resynchronization therapy; CS = coronary sinus; EDD = end-diastolic diameter; ESD = end-systolic diameter; LA = left atrium; LVEF = left ventricular ejection fraction; MR = mitral regurgitation; NYHA = New York Heart Association.

## Data Availability

The datasets used in the current study are available from the corresponding author upon reasonable request.
